# High Protein Substitutes for Gluten in Gluten-Free Bread

**DOI:** 10.3390/foods10091997

**Published:** 2021-08-25

**Authors:** Adriana Skendi, Maria Papageorgiou, Theodoros Varzakas

**Affiliations:** 1 Department of Food Science and Technology, International Hellenic University, P.O. Box 141, 57400 Thessaloniki, Greece; andrianaskendi@hotmail.com; 2 Department of Food Science and Technology, University of the Peloponnese, 24100 Kalamata, Greece; t.varzakas@uop.gr

**Keywords:** gluten-free bread, plant proteins, animal proteins, microalgae, optimized bread structure, protein substitutes

## Abstract

Gluten-free products have come into the market in order to alleviate health problems such as celiac disease. In this review, recent advances in gluten-free bread are described along with plant-based gluten-free proteins. A comparison with animal-based gluten-free proteins is made reporting on different high protein sources of animal origin. Sea microorganisms- and insect-based proteins are also mentioned, and the optimization of the structure of gluten-free bread with added high protein sources is highlighted along with protein digestibility issues. The latter is an issue for consideration that can be manipulated by a careful design of the mixture in terms of phenolic compounds, soluble carbohydrates and fibres, but also the baking process itself. Additionally, the presence of enzymes and different hydrocolloids are key factors controlling quality features of the final product.

## 1. Introduction

There is an increasing number of people that sufferhealth problems from the consumption of wheat and other cereals that contain gluten as well as all their derived products. They can experience different health conditions such as celiac disease, non-celiac gluten sensitivity (NCGS), wheat allergy or irritable bowel syndrome [1].

Celiac disease is a specific immune response that is caused after consumption of gluten present in wheat, rye, barley and related grains from genetically predisposed patients. On the other hand, wheat allergy is triggered by the consumption of insoluble gliadins of wheat that react with immunoglobulin E (IgE) causing allergy symptoms that could be life-threatening. Contrary to celiac disease, wheat allergy is not reported to cause permanent damage to the gastrointestinal system [1,2]. For celiac patients and those diagnosed with wheat allergy, the only treatment is to adhere to a very strict diet that does not contain gluten or a wheat-free diet, respectively.

According to Catassi et al. [3], NCGS is a syndrome observed in patients that are not affected by either celiac disease or wheat allergy and are characterized by intestinal and extra-intestinal symptoms due to the ingestion of foods containing gluten. In their review, Barbaro et al. [4] and Biesiekierski and Iven [1] report that is still not clear if is the gluten or other wheat components that cause NCGS, but at the same time, they suspected that its prevalence to be higher than that of celiac disease. Thus, patients diagnosed with NCGS have to eliminate gluten from their diet to some extent. The difference with other celiac and allergic patients lies in the extent to which each one can tolerate the gluten depending on the severity of the symptoms he experiences.

The population affected by the celiac disease, that by necessity has to consume only gluten-free products, was estimated to be approximately 1% of the total Western population with some Western European populations showing higher prevalence [2]. On the other hand, nowadays, gluten-free foods are not consumed only by those consumers experiencing digestive problems with gluten or wheat. Specifically, another group of consumers exists that deliberately avoids or limits gluten as a part of a diet regime or other expected health benefits. Thus, the demand for gluten-free products is high and besides this, there is foreseen a continuous increase in the trade of gluten-free products reaching 8.3 billion US dollars in 2025 [5].

The food industry is focusing on the production of gluten-free products in order to fulfil this increasing need. Among the gluten-free products that are produced, bread occupies a special place. Conventional wheat bread, or bread made with other cereals such as rye, barley and oat, represents a staple food present on a daily basis in the table of the consumers. Eliminating bread constitutes a great deprivation for those following a gluten-free diet. Therefore, the production of good quality and tasty gluten-free bread represents a major challenge for the bakery industry.

The recipe for gluten-free bread varies depending on the gluten-free ingredients used. In the market, there are circulating different gluten-free mixtures. The most common wheat flour substitutes for the production of gluten-free bread are rice and/or maize flours combined with starch of different origins (e.g. potato, corn, cassava). These ingredients are the most abundant and the cheapest. Gluten-free mixtures are mainly composed of carbohydrates and lack in protein content. The latter not only affects the required daily dietary amount of proteins but also largely affects the bread structure and quality. In conventional wheat bread, the open cellular structure is due to the elasticity of the gluten that after mixing with water is able to entrap carbon dioxide (CO_2_) produced by yeasts during fermentation in the leavened dough, causing the dough to rise. Although being a minor component of gluten, the glutenin macro polymer (GMP) fraction is considered the main contributor of the elastic properties observed in the wheat dough, playing an important role in breadmaking [6].

Besides, the improvement of the nutritional profile of gluten-free products has become an important target due to a low protein and fibre content and a high fat and salt content raising health issues to consumers suffering from other diseases such as cardiovascular diseases or diabetes. However, during the last years, efforts have been made to improve this, e.g., the level of dietary fibre content [7,8]. A gluten-free bread that is made of maize flour instead of wheat flour is also considered low in the amino acids lysine and tryptophan and high in other large amino acids such as leucine and valine [9]. Therefore, the amino acid profile must also be considered.

Since gluten, which is responsible for obtaining raised bread loaves, is missing in gluten-free breads, its structure deteriorates from that of conventional bread. It is difficult to mimic the properties of gluten with other proteins. A great number of studies have tried to improve both the quality and the nutritional profile of gluten-free bread by increasing protein content using appropriate protein concentrates or isolates obtained from microorganisms, animals and plants. According to Akharume et al. [10], protein ingredients in commerce fall into three categories; protein flours, protein concentrates and protein isolates that contain 10–20%, 55–60% and more than 80% protein content, respectively. Gorissen et al. [11] have monitored protein content and amino acid composition of some of the commercially available plant-based protein isolates such as oat, lupin, wheat, hemp, microalgae, soy, brown rice, pea, corn, potato, milk, whey, caseinate, casein, egg and human skeletal muscle protein. They observed that the content of essential amino acids of plant-based protein isolates was lower than that of animal-based proteins. In addition, the profile of amino acids differs, with methionine and lysine being higher in animal-based proteins. They suggest the use of a balanced combination of different plant-based proteins in order to increase the quality of protein in the blend.

The literature reports the development of a range of gluten-free breads enriched with alternative sources for protein trying to improve the quality and taste of the final product. In a search with keywords “gluten-free AND protein AND isolate” “gluten-free AND protein AND concentrate” present in the article title, keywords and abstract, the scopus database returned 74 and 53 total results, of which 45 and 32, respectively, were published from 2016 until now (June 2021). Thus, this subject represents an issue that attracts researchers’ interest during these last five years. In this review, we gathered published data and report recent advances made in the development of gluten-free products with high protein substitutes for gluten, with the main focus on bread.

## 2. Plant-Based Gluten-Free Protein

Many studies found in the recent literature report the use in a gluten-free bread recipe of highly concentrated protein sources of plant origin (Table 1). The use of these sources intends to improve not only bread quality but also its nutritive values. Using protein-rich sources of plant origin has gained interest due to the limitation of animal origin counterparts due to their allergenic character. Generally, the gluten-free flours of plant origin differ in the content and quality of protein. Wu et al. [12] prepared breads from a range of gluten (white wheat, wholemeal wheat, spelt and rye) and gluten-free (lupin, buckwheat, chickpea, amaranth) flours standardized at 10% protein with maize starch. They observed differences in the proportions of essential amino acids (0.31–0.35 and 0.34–0.41 for gluten and gluten-free flours, respectively) with lysine and arginine showing much higher levels in gluten-free flours compared to glutenous flours, whereas the opposite was observed for proline. Low levels of proline in gluten-free flours were considered responsible for the lower rising capacity during proofing.

In recent years, different plant-based high protein isolates have been used for gluten-free bread preparation (Table 1). Rice flour is considered as the most suitable among the cereal flours for the production of gluten-free bread because it is considered hypoallergenic and has high digestibility besides a bland taste and white colour that do not affect the final bread quality. Derived from rice flour, rice protein isolate is used as a safe source of protein in gluten-free bread production [13]. On the other hand, rice bran protein concentrate obtained from rice bran that is a by-product of rice production is also considered a non-allergenic protein. It is utilized for increasing the protein content of gluten-free bread [15]. It is isolated from rice bran via an alkaline-acid extraction technique resulting in 68% protein on a dry basis. Zein, a protein from another non-gluten cereal, maize, was used to provide extensibility to starch-based doughs [16,17] and firmness to the bread crumb comparable to wheat breads [18].

Besides non-gluten cereals, proteins from legumes have attained the interest of researchers due to their adequate protein profile. Although being high in lysine, they are deficient in amino acids methionine, cystine and cysteine [28].

Soy protein isolate is obtained by extraction from the soy bean, and is of high biological value due to high amounts of the essential amino acids lysine and methionine [29]. It has the ability to alter water absorption of the dough mixture and thereby impacts its rheology. Moreover, soy protein isolates have high foaming capacities, as well as high foam stability [20]. Contradictory results are reported in the literature about the resulted bread volume and crumb structure [20,22]. Horstmann, Foschia and Arendt [20] report that soy-protein-enriched bread (2% on potato starch) produces bread with a low volume and a dense crumb structure and a low consumer acceptance score, whereas Masure, Wouters, Fierens and Delcour [22] showed that the addition of soy (4% on rice flour) produced bread with a similar volume of that of the control bread made of rice flour and in a homogeneous crumb structure with very large gas cells.

Lupin protein represents another protein-rich source gaining interest in gluten-free bread production [19]. It was noted that the extraction method used to obtain lupin protein concentrates/isolates plays a significant role in the functional qualities of lupin proteins, including their chemical composition, emulsification, rheology and thermal properties [30].

Pea protein, an extract from pea seeds, has become a popular product in the food industry due to its well-balanced amino acid profile rich in the essential amino acid lysine [31]. According to Horstmann, Foschia and Arendt [20], the emulsifying capacity of legume isolates decreases in the following order: soy > lupin > pea, whereas emulsifying stability decreases in the order: lupin protein > soy protein > pea protein.

**Figure 1:**
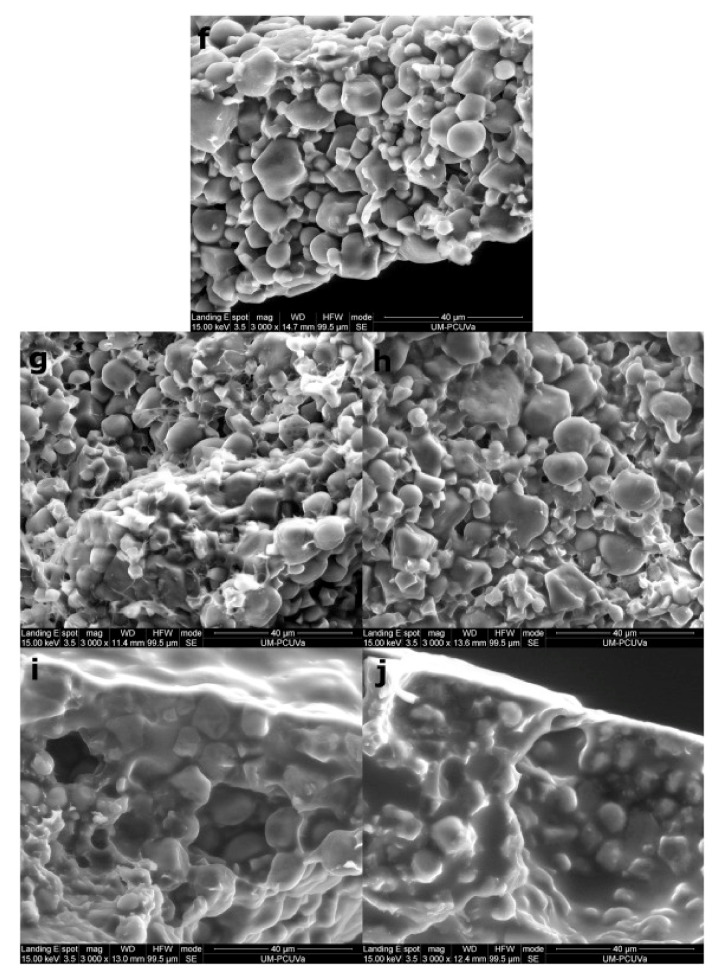
Micrographs of crust cross-section at 3000× magnification. Images correspond to breads supplemented with 10% protein and control sample: Control (50% rice flour: 50% maize starch) (**f**), Rice protein 10% (**g**), Pea protein 10% (**h**), Egg white powder 10% (**i**), Whey protein 10% (**j**) [14].

A highly concentrated protein of legume origin is being used recently not only to equilibrate the amino acid profile of gluten-free bread but also to strengthen the protein matrix in these breads. Micrographs in Figure 1 reveal the matrix created in the gluten-free breads with different protein sources. Compared to the control bread made of rice flour and maize starch (Figure 1f), cross-sectional photographs of gluten-free bread with added rice and pea protein (Figure 1g and h, respectively) show a higher number of small filaments connected to starch granules. These filaments are more evident for rice protein and represent protein molecules that link starch granules [14]. Incorporation of rice protein until 10% increases the specific volume of the gluten-free bread whereas when added 5% of pea proteins, there is not observed any difference with the control. Any further increase (10%) decreased the specific volume of the bread [14]. In their study, Ziobro, Juszczak, Witczak and Korus [19] observed that the pea and soy protein addition at 10% level has a negative effect on bread volume, whereas lupine protein showed similar values with control bread (maize and potato starch). The crumb structure of pea, soy and lupine breads was more porous and has more large pores than control bread. Nevertheless, lupine is considered a better option due to the lower hardness of crumb than pea and soy.

The incorporation of legume proteins produces gluten-free breads with darker crumb and crust [13]. Among proteins of vegetal origin added to gluten-free bread, pea protein decreases the luminosity of the bread crust to a greater extent than rice, due to the higher lysine content that reacts with carbohydrates producing more coloured products during the Maillard reaction [13]. Although different flavours are produced during the baking of breads there are not all considered attractive to the consumers, it was observed that the acceptability of panellist decreases in the following range: lupine protein, pea protein, soy protein.

Different regulatory authorities such as European [32], Australian and New Zealand [33] legislation consider rapeseed protein isolate (containing 96% on dry basis protein) as a novel food ingredient for use in food products, whereas the US FDA gives GRAS status [34]. Its inclusion in the gluten-free bread recipe provides not only valuable amino acids but also affects the pasting characteristics of starch and modifies rheological characteristics of dough [24] as well as improves quality characteristics, sensory attributes and storage of gluten-free bread [23]. Levels of addition higher than 9% resulted in higher bread volume and lower hardness of the breads during storage compared to control bread. In another study, when canola proteins were added to a white rice flour bread recipe up to 6% resulted in an improvement of the technological properties of the dough and the resulted bread [25].

Sunflower protein concentrate represents another source of protein. Containing more than 75% proteins, it is obtained by extraction from the cake that remains after oil extraction from the seeds. Its high water-holding capacity decreased bread hardness during the storage, whereas the dark colour decreased the brightness of the bread making them more attractive to the panellist due to the similarity with whole flour [26].

Potato protein is extracted from the remains after the removal of starch [35]. The nutritional quality of potato protein is considered high in quality because it contains a high proportion of lysine, approaching the quality of proteins in eggs [36]. This isolate (>90% protein dry base) was employed by Witczak, Juszczak, Ziobro and Korus [27] for the production of gluten-free bread.

## 3. Animal-Based Gluten-Free Protein

Among the high protein sources of animal origin, those based on milk and eggs are widely used to increase the protein content and improve amino acids’ profile in gluten-free breads. These characteristics made these sources highly popular for gluten-free breads in the recent scientific literature (Table 2). Proteins of animal origin have good solubility, high emulsifying and foaming capacity and high stability. This fact is observed in surface photographs (Figure 1) where a film is visible covering the starch granules in the case when egg white powder and whey protein were used. There is observed a very distinct difference when compared to the control bread or to breads with rice and pea protein isolates [14]. Animal proteins such as whey protein and egg white powder were found to decrease the crispness in the crust of gluten-free breads [14]. The type of protein added affects the crust colour since it reacts with the carbohydrates triggering Maillard reactions. In general, when protein sources of animal-origin are added to a gluten-free recipe, they decrease the luminosity of crust, producing breads with a darker and more reddish crust than the control bread [13,19,37]. There are differences in the decrease in luminosity among the different protein sources, with whey protein yielding a darker crust than egg white powder due to the high lysine content that this protein contains and the importance it has in the Maillard reaction [14]. In the literature, there are reported contradictory results for the crust and crumb colour, possibly due to the differences in the level of proteins added [13,15,19,37]. Generally, the higher the protein addition level, the darker the colour [37].

The volume and the texture parameters of the breads with added animal protein content vary depending on the protein type and the level of addition [13,15,19,38,39]. For example, Ziobro, Juszczak, Witczak and Korus [19] report an increase in the specific volume of breads when 10% albumin was added. Contrarily, Phongthai, D’Amico, Schoenlechner and Rawdkuen [15] report an increase in the specific volume of bread when the level increases to 2% and then a decrease for a further increase to 4%, whereas Sahagún and Gómez [13] report a decrease at the 30% addition level. A comparison is difficult since in the above studies, the egg protein source was added in different flour mixtures, and different levels of hydration were applied resulting in different dough systems. The same is valid for the texture and the structure of these breads. Moreover, the composition of the high protein source can affect the quality of the bread. Han, Romero, Nishijima, Ichimura, Handa, Xu and Zhang [39] compared two egg white sources of similar composition but differing in water solubility and reported that the powder with more water-soluble protein aggregates was associated with larger improvement in bread quality. On the other hand, Masure, Wouters, Fierens and Delcour [22] reported that regular egg white powder and dry heated egg white powder produced similar bread loaf volume, but regular egg white showed lower firmness during storage than the dry heated counterpart. Another factor that must be taken into consideration is the basic flour mixture used to prepare the gluten-free bread. In their study, Aprodu and Banu [38] observed that the type of starch also affects the efficiency of whey protein on thermo-mechanical properties, specific volume and firmness of the bread crumb.

Collagen is reported as more effective than albumin to reduce the hardness and prevents staling of gluten-free breads [19]. Generally, the hardness of the crumb is increased with increasing protein concentration [37], suggesting the need for optimization of the level of protein enrichment.

## 4. Sea Microorganisms- and Insect-Based Proteins

Another protein source used for the preparation of gluten-free breads can be obtained from algae, seaweed and insects (Table 3). Seaweeds or macroalgae are complex multicellular organisms that grow in salt and marine environments, and most of them can be used for direct human nutrition.

Microalgae are considered as a rich source of protein of high quality (rich profile of essential amino acids), besides other bioactive compounds (e.g., polyunsaturated fatty acids, carotenoids, vitamins) [47]. Becker [47] in his review reports that the protein content of different algae that can be used in the food industry varies 6–71% of the dry matter, but most of them contain more than 28%. In addition, the amino-acid profile of microalgae protein is considered well-balanced and comparable with that recommended by WHO/FAO and that of eggs and soybean [47]. Some varieties of macroalgal species have been used to obtain bioactive peptides that exert beneficial health effects beyond those benefits associated with basic nutritional values [48].

Among microalgae, Chlorella species were reported to have a high amount of protein in combination with an adequate amount of essential amino acids, especially higher levels of the essential amino acids lysine and tryptophane, in order to provide adequate nutrition [49]. Besides protein, they contain in high amounts carbohydrates (8–64%) and lipids (2–22%) [47]. In order to minimize production cost, instead of refined protein, the industry tries to promote the use of algal biomass as a whole in powder form and not as a protein isolate. The use of these powders was reported to alter sensory characteristics of the bread such as colour, aroma and flavour as well as texture [40,41]. Breads enriched with C. sorokiniana were characterised by low luminosity and a deep green colour [40]. Similarly, other authors observed a decrease in bread luminosity and an increase in green and yellow colour when two other microalgae were used as the species, observing differences that are due to both species and level of addition [41]. Changes in dough and bread colour are due to the presence of pigments (mainly chlorophyll) in microalgal biomass.

In their study, Khemiri, Khelifi, Nunes, Ferreira, Gouveia, Smaali and Raymundo [41] evaluated the effect of the incorporation of two different microalgae (*Nannochloropsis gaditana L2* and *Chlamydomonas* sp. EL5) on the dough properties. They suggest that for levels 1% and 3%, there was no need to adjust the water content and observed that dough mixing curves of microalgae-added doughs were similar to that of the control. The incorporation of microalgae increased dough development time and stability of the doughs, but differences were noticed based on microalgae strains and the level of addition. On the other hand, texture parameters (firmness, adhesiveness and cohesiveness) of the doughs were not affected by microalgae addition. Contrarily, the addition of microalgae significantly increased the firmness and adhesiveness of the gluten-free bread crumb as microalgal biomass incorporation increased, but without producing significant changes in bread cohesiveness. These changes were considered positive by the authors since it helped to strengthen the texture of the gluten-free bread by reinforcing the protein structure of the bread and reduce the brittleness that characterizes gluten-free breads [41]. In another study, breads with microalgae *C. sorokiniana* at a higher level of addition (4.2%) showed increased crumb porosity in comparison with the control bread. This behaviour was attributed to the high protein and lipid contents present in the powder [40]. Generally, besides the odour, taste and texture, panellists appreciated the intense green colour of the 3% supplemented bread scoring higher than the control [41].

Dough and bread pH is a parameter of great importance since it determines the growth kinetic of yeasts/microorganisms during fermentation, and the final bread pH is linked with bread taste and storage but often is not reported. The addition of microalgal biomass increased the pH of the gluten-free dough with values varying from 5.77 in control to 6.05 and 6.01 for *Chlamydomonas* sp. EL5 and *N. gaditana* L2, respectively, when the microalgae were added at 3% [41]. Similarly, Różyło, Hameed Hassoon, Gawlik-Dziki, Siastała and Dziki [43] observed an increase in the pH of dough that ranged from 5.05 in control dough to 5.2 in the dough with 10% brown algae added. These values differ due to the different initial mixture used to prepare the gluten-free bread as well as the composition and level of the microalgae added. The addition of brown algae increased the bread volume [43]. The authors suggested that algae components are hydrated, swelled and gelatinized at a slower rate compared to the control flour mixture. Moreover, they report that it was due to the algal protein enrichment that improved the rheological properties of dough and increased the gas retention capacity of the dough that resulted in higher bread volume. Since algae contain pigments, their addition decreased the lightness of bread crumb. The brown algae addition decreased bread firmness and the staling rate, whereas the bread elasticity was increased with algae addition. Nevertheless, sensory tests demonstrated that the addition of much less amount (2%) of brown algae can lead to an acceptable gluten-free bread.

Spirulina was another microalgae used to enhance the protein content by 20% in rice bread when its level was increased from 1 to 4% [42]. Besides the decrease in the brightness of bread with increasing spirulina level, an increase in the specific volume of the bread was observed. Dough elasticity influences gas retention capacity and the specific volume of breads. Panellists preferred gluten-free bread with 1% content of spirulina than that with 4% because of the lighter crumb colour, although the general score was similar for breads with 1 and 4% spirulina.

Red seaweed (*Palmaria palmata)* contains 9–26% protein, and the amount of vital amino acid lysine can reach 5.9 g/100 g of total amino acids [50]. Hydrolyzation of a protein extract from *P. palmata* with food-grade enzymes released renin inhibitory peptides [51,52]. The protein hydrolysates from *Palmaria palmata* rich in inhibitory peptides that were used to increase the nutritional profile of wheat bread [51] could be used as an alternative protein source.

Besides microalgae, insects have attracted researchers as a new source of protein since they can contain more than 40–75% of their dry base. In addition, their protein is 77–98% digestible from the human organisms and rich in (46–96%) essential amino acids [53]. Among the insects, the Orthoptera (Grasshoppers and locusts)were found to have the higher amount of proteins (61–77) [53].

One study where cricket powder (*Acheta domesticus*) was used to replace starch (from rice and corn) at levels 2%, 6% and 10% resulted in exceeding a two-, four- and seven-fold increase in nutritional value, in terms of the protein content, compared to control bread [44]. In addition, a significant decrease in crumb lightness was observed. This replacement induced changes at the molecular level, stabilizing water transport, that delayed the process of bread staling and resulted in reduced bread hardness [54]. In another recent study [45], where the cricket powder was used for the fortification of gluten-free mixture made of rice and maize flour with protein at about 5.5%, there was observed that the bread developed a unique bouquet of volatile organic compounds. The aroma compounds developed were considered similar to those of the reference dough regarding acetoin and acetate, slightly higher ethanol and lactate and a little lower 1,4-butanediol content. In addition, cricket-powder-enriched samples developed a typical flavouring profile made by nuances of hexanoic and nonanoic acid, 2,4-nonadienal, 1-hexanol, 1-heptanol, and 1-octen-3-ol, 2,4-butanedione, 2-heptanone and 3-octen-2-one. Besides this, cricket powder inclusion in the gluten-free recipe increased the amount of soluble proteins in cricket dough compared to the reference dough.

Another type of cricket powder (*Gryllus assimilis*) was used at the 10 and 20% level of addition in a mixture of rice flour and maize starch to improve the protein content of the gluten-free bread [46]. The authors observed that enrichment with cricket powder increased the hardness of the bread due to the formation of a more stable structure compared to the control. Nevertheless, when added at the same levels with lentil and buckwheat flour, the increase in the hardness derived from the cricket powder was less. Moreover, when compared to lentil and buckwheat flour, as a protein source, cricket flour was more efficient to improve the cohesiveness and the springiness of the breads. Bread with high cohesiveness keeps its integrity during slicing and mastication whereas that with high springiness has the ability to return to its original shape after compressing. Moreover, due to higher protein content, breads with cricket powder have higher porosity compared to respective breads made with lentil and buckwheat flour. The authors reported that in order to obtain the best in terms of bread quality, no oil should be used in the case of cricket powder since it is rich in lipids.

## 5. Optimizing the Structure of Gluten-Free Bread with Added High Protein Sources

It is advisable to use transglutaminase (TGase) in gluten-free bread containing added protein sources because it helps the creation of a protein network in the gluten-free dough [27,42,55,56,57]. The simultaneous addition of TGase with protein was reported to be more effective than adding them separately [57]. Transglutaminase is an enzyme that forms ɛ-(γ-glutamyl) lysine cross-links when acting with proteins [58], affecting protein properties such as water-holding capacity, gelation capability, thermal stability, etc. This behaviour is utilized to promote a protein network that in its turn improves viscoelastic properties of the gluten-free dough. The amount and the enzyme, its origin as well as the protein substrate largely affect the efficiency of the structure created [59]. Selmo and Salas-Mellado [42] reported that when the concentration of the protein source (spirulina) is high, the amount of transglutaminase should be less in order to increase the specific volume and decrease the firmness of the gluten-free bread. In a previous study, Dłużewska, Marciniak-Lukasiak and Kurek [56] reported that higher levels of TGase (10U/g protein) negatively affected the texture, staling and sensory characteristics of gluten-free breads with soy and whey protein isolate. Moreover, they observed that microbiological TGase was more efficient in increasing the specific volume and crumb porosity of gluten-free bread with soy protein isolate compared to whey protein isolate [56]. In another study, when the enrichment of rice flour with three different protein sources (soy, casein and whey protein isolate)was studied, the addition of TGase further increased the specific volume of bread, reduced the second proofing time and decreased the hardness of the crumb [57]. On the contrary, the findings of Moore, Heinbockel, Dockery, Ulmer and Arendt [59] reported skim milk powder and egg powder but not soya flour as a good substrate for TGase, which allowed the creation of substantial protein networking that improved loaf volume, crumb characteristics and the appearance of gluten-free breads.

In order to obtain gluten-free bread with a high specific volume, it is of great importance to adjust the hydration of the doughs based on the final selected recipe [60]. Especially when a high protein source is added, it will alter the water distribution among the recipe components. This variation will be dependent on the difference in water-binding capacities of proteins in the recipe. The ability of proteins to absorb water affects dough rheology and bread volume. Contrary to the wheat-based dough, the amount of water in the case of gluten-free breads cannot be calculated based on farinograph consistency. Baking tests of gluten-free breads prepared with the stepwise increase/decrease of water are generally needed. Nevertheless, it was observed that more water is needed to be used in a bread recipe with vegetal proteins than the breads with animal proteins to obtain maximum values of specific volume [13]. Water levels of 150% and 115% are needed when pea and rice protein are added at the 30% level, compared to 90% needed for the control (100% maize starch). For the same level of addition, the animal proteins egg white and whey protein needed 85% and 40% water, respectively. Although, Sahagún and Gómez [13] and Bravo-Núñez et al. [61] optimized the hydration level of bread, they use a very high level of protein source (30%) that can be blamed for the negative effect observed in bread specific volume and texture. Thus, besides the moisture content, optimization of the addition level depending on the basic flour mixture is also needed.

Besides enzymes, the addition of hydrocolloids was found to be very effective in order to improve the gluten-free bread structure when a rich source of protein is added. Hydrocolloids have the ability to increase the viscosity and water holding ability of the dough system due to high molecular weight and help to create a more stable structure.

Xanthan gum is reported to be used together with rich protein for better results [56,59]. In these studies, the optimal recommended amounts of xanthan vary from 0.75 to 1.5 to be added no matter what type of protein was used (skim milk powder, soy flour, egg powder, soy protein isolate, whey protein isolate). Moreover, methylcellulose is another hydrocolloid used in gluten-free mixtures together with rich protein sources [42]. Selmo and Salas-Mellado [42] reported that optimal levels to be used are above 1.5% but less than 2.2%. The simultaneous addition of proteins and hydroxypropyl methylcellulose (HPMC) improves the porosity of gluten-free bread, being similar to that of wheat bread [21]. According to Manik and Nur [62], HPMC and xanthan gum are considered the most suitable hydrocolloids for a gluten-free bread with the first being more effective in increasing the volume of the bread and producing a softer crumb.

**Figure 2:**
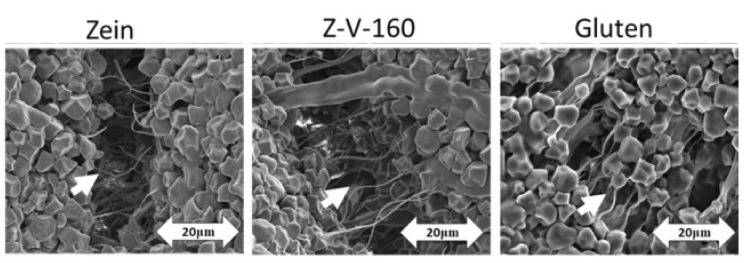
Images of dough samples containing starch with zein, thermally-treated-zein (Z-V-160) and gluten [16].

In addition, the extraction procedure, as well as a treatment performed on the protein source, could affect the properties of proteins that are of great importance for the dough behaviour and final bread quality. Zein’s limited ability to improve dough extensibility was surpassed by applying thermal treatment [16] and extrusion [17]. These treatments improved the gluten-free dough structure. In Figure 2, one can visualize the microstructure of the dough during extension [16]. Dough samples with rice starch and untreated zein showed a higher number of broken fibrous linkages during stretching that was not observed in thermally treated zein at 160 °C (Z-V-160) or in samples with added wheat gluten, as indicated by white arrows. This suggests that thermally treated zein was able to create a more similar structure to gluten compared to the untreated one, and the thermal treatment could further improve the quality of bread.

From the abovementioned studies, one can conclude that the right combination of the flour source rich in carbohydrates, protein source and hydrocolloids/enzymes in addition to the right hydration level and possible protein isolate treatments could play an important role in the optimal structure-forming of breads.

## 6. Protein Digestibility

Simonato et al. [63] reported that protein-specific immune responses in people with wheat “allergy” were due to reactivity caused primarily to baked crumb and crust and not to dough. Thus, enhancing protein digestion techniques can lower the allergenic effects of gluten proteins. Wu, Taylor, Nebl, Ng and Bennett [12] observed differences between the gluten and gluten-free flours in terms of digestibility and size distribution of undigested peptides. Gluten-free products (pre-mix, doughs and breads) differ in their counts of total undigested peptides from the respective gluten products; in gluten containing products, they remained constant for pre-mix to baked breads while in non-gluten ones, digestibility was increased significantly during proofing but remained unchanged during baking.

Digestibility of bread proteins is affected by the interaction of macro-nutrients and micro-nutrients during all stages of bread preparation such as mixing, proofing and baking. During baking, digestibility of proteins is decreased due to the denaturation and reaction between neighbouring proteins and carbohydrates (reducing sugars) [64]. Phenolic compounds were considered a significant factor that affected protein digestibility [12,65,66,67] and in the second place were listed the carbohydrates [12]. Phenolics affect recognition sites of digestive enzymes during mixing and mediate protein-protein cross-linking and thus decrease digestibility. On the other hand, carbohydrates (reducing sugars) responsible to trigger Maillard reactions (mostly at the surface or the bread crust) with proteins contribute to a lesser extent, especially during moderate baking conditions [12]. Besides the aforementioned factors, the presence of some micro-compounds such as enzyme inhibitors or phytic acid present in some of the gluten-free flours affect protein digestibility of breads since proteins are bound by phytic acid in insoluble binary and ternary structures, making them unavailable for digestion [68].

In their study, Wu, Taylor, Nebl, Ng and Bennett [12] suggest an appropriate design of formulation in order to improve protein digestion and increase protein availability. The increased ratio of soluble carbohydrate to protein, limiting the amount of fibre and phenolics, could improve protein digestion, but these changes affect negatively other dietary components of bread (antioxidants and fibre content). On the other hand, maximizing gas production and retention during fermentation could limit the opportunity for protein cross-linking since it helps to maximize the separation of parallel protein strands. In addition, modification of baking conditions (moisture heating) could modify protein digestibility. Thus, the availability of proteins can be increased by applying a careful design of the mixture as well as the baking process.

## 7. Conclusions

In this review, plant-based gluten-free proteins are compared to animal-based gluten-free proteins, with the main focus on gluten-free bread. Protein digestibility issues are very important in this context and should be taken seriously into account. Moreover, the optimization of the structure of gluten-free bread with added high protein sources should be considered along with factors such as hydration, the protein extraction procedure, viscosity and water holding ability of the dough system. The presence of enzymes and different hydrocolloids are key factors controlling the above parameters, while the digestibility of the added protein is also an issue for consideration that can be manipulated by a careful design of the mixture in terms of phenolic compounds, soluble carbohydrates and fibres but also the baking process itself.

## Figures and Tables

**Table 1:** Different high protein sources of vegetable origin reported in recent published literature to improve gluten-free bread.

Source	Concentration (% in the Starchy Flour Mixture)	Control Bread	Literature
**Gluten-free cereals**			
rice protein	30%	100% maize starch	[13]
rice protein	5%, 10%	50% rice flour: 50% maize starch	[14]
rice bran protein concentrate	2%, 4%	100% rice flour	[15]
Zein	15%	15% vital wheat gluten: 85% rice starch	[16]
Zein	15%	100% wheat flour, 100% starch from: rice, maize, potato	[17]
Zein	2.5%, 5%, 10%	100% wheat flour, ~88% maize starch: ~12% potato starch	[18]
**Legumes**			
Pea protein	30%	100% maize starch	[13]
Pea protein	5%, 10%	50% rice flour: 50% maize starch	[14]
Pea protein	~10%	80% maize starch: 20% potato starch	[19]
Pea protein	2%	100% potato starch	[20]
Lupin protein	~10%	80% maize starch: 20% potato starch	[19]
Lupin protein	2%	100% potato starch	[20]
Soy protein	~10%	80% maize starch: 20% potato starch	[19]
Soy protein	2%, 4%, 6%	100% rice flour	[21]
Soy protein	4%	100% rice flour	[22]
Soy protein	2%	100% potato starch	[20]
**Oil seeds**			
Rapeseed protein	6%, 9%, 12%, 15%	80% corn starch: 20% potato starch	[23]
Rapeseed protein	6%, 9%, 12%, 15%	80% corn starch: 20% potato starch	[24]
Canola protein extract	3%, 6%, 9%	100% wheat flour, 100% rice flour	[25]
Sunflower protein	5%, 10%, 20%	70% rice flour: 30% maize starch	[26]
**Tubers**			
Potato protein	2%, 6%, 10%	80% maize starch: 20% potato starch	[27]
Potato protein	2%	100% potato starch	[20]

**Table 2:** Different high protein sources of animal origin in recent published literature to improve gluten-free bread.

Source	Concentration (% of Starchy Flour Mixture)	Control Bread	Literature
Dairy			
whey protein	10%, 20%, 30%	100% wheat flour, 50% cassava starch: 50% chickpea flour	[37]
whey protein	30%	100% maize starch	[13]
whey protein	5%, 10%	50% rice flour: 50% maize starch	[14]
whey protein	2%, 4%, 6%	100% rice flour	[21]
whey protein	12% *	50% quinoaflour: 50% (maize starch, potato starch, modified maize starch, modified potato starch)	[38]
**Eggs**			
egg white powder	30%	100% maize starch	[13]
egg white powder	5%, 10%	50% rice flour: 50% maize starch	[14]
egg white powder	2%, 4%	100% rice flour	[15]
egg white powder	~10%	80% maize starch: 20% potato starch	[19]
egg white powder	5%, 10%, 15%	Commercial gluten-free flour (mixture of garbanzo bean flour, potato starch, tapioca flour, whole grain sorghum flour and fava bean flour)	[39]
egg white powder	4%	100% rice flour	[22]
**Other animal sources**			
collagen	~10%	80% maize starch: 20% potato starch	[19]

* the added protein amount is calculated based on the total flour mixture, and its addition is associated with removal of only the same amount of starch fraction.

**Table 3:** Different high protein sources from algae and insects reported in recent published literature to enrich gluten-free breads.

Source	Concentration (% of Starchy Flour Mixture)	Control Bread	Literature
**Algae**			
Chlorella powder (*Chlorella sorokiniana*)	2.1%, 4.2%	25% rice flour: 58.3% maize starch: 16.7% pea flour	[40]
Microalgae powder (*Nannochloropsis gaditana* L2; *Chlamydomonas* sp. EL5)	1%, 3%	31% rice flour: 46% buckwheat: 23% potato starch	[41]
Spirulina (strain LEB -18)	1–4%	100% rice flour	[42]
Brown algae powder (*Ascophyllum nodosum*)	2%, 4%, 6%, 8%, 10%	45% white rice flour: 45% maize flour: 10% millet flour	[43]
**Insects**			
Cricket powder (*Acheta domesticus*)	2%, 6%, 10%	80% maize starch: 20% potato starch	[44]
Cricket powder (*Acheta domesticus*)	5.5%	80% maize flour: 20% rice flour	[45]
Cricket powder (*Gryllus assimilis*)	10%, 20%	70% rice flour: 30% maize starch	[46]

## Abbreviations

Non-celiac gluten sensitivity (NCGS); Carbon dioxide (CO_2_); glutenin macro polymer (GMP); hydroxypropyl methylcellulose (HPMC); immunoglobulin E (IgE); Total Nitrogen (TN), Transglutaminase (TGase); World Health Organization (WHO).
